# Who will lose weight? A reexamination of predictors of weight loss in women

**DOI:** 10.1186/1479-5868-1-12

**Published:** 2004-08-02

**Authors:** Pedro J Teixeira, António L Palmeira, Teresa L Branco, Sandra S Martins, Cláudia S Minderico, José T Barata, Analiza M Silva, Luís B Sardinha

**Affiliations:** 1 Department of Exercise and Health, Faculty of Human Movement – Technical University of Lisbon, Cruz Quebrada, PORTUGAL

## Abstract

**Background:**

The purpose of this study was to analyze pretreatment predictors of short-term weight loss in Portuguese overweight and obese women involved in a weight management program. Behavioral and psychosocial predictors were selected a priori from previous results reported in American women who participated in a similar program.

**Methods:**

Subjects were 140 healthy overweight/obese women (age, 38.3 ± 5.9 y; BMI, 30.3 ± 3.7 kg/m^2^) who participated in a 4-month lifestyle weight loss program consisting of group-based behavior therapy to improve diet and increase physical activity. At baseline, all women completed a comprehensive behavioral and psychosocial battery, in standardized conditions.

**Results:**

Of all starting participants, 3.5% (5 subjects) did not finish the program. By treatment's end, more than half of all women had met the recomended weight loss goals, despite a large variability in individual results (range for weight loss = 19 kg). In bivariate and multivariate correlation/regression analysis fewer previous diets and weight outcome evaluations, and to a lesser extent self-motivation and body image were significant and independent predictors of weight reduction, before and after adjustment for baseline weight. A negative and slightly curvilinear relationship best described the association between outcome evaluations and weight change, revealing that persons with very accepting evaluations (that would accept or be happy with minimal weight change) lost the least amount of weight while positive but moderate evaluations of outcomes (i.e., neither low nor extremely demanding) were more predictive of success. Among those subjects who reported having initiated more than 3–4 diets in the year before the study, very few were found to be in the most successful group after treatment. Quality of life, self-esteem, and exercise variables did not predict outcomes.

**Conclusions:**

Several variables were confirmed as predictors of success in short-term weight loss and can be used in future hypothesis-testing studies and as a part of more evolved prediction models. Previous dieting, and pretreatment self-motivation and body image are associated with subsequent weight loss, in agreement with earlier findings in previous samples. Weight outcome evaluations appear to display a more complex relationship with treatment results and culture-specific factors may be useful in explaining this pattern of association.

## Background

Predicting weight loss outcomes from information collected from subjects before they start weight management programs is a long-standing goal [1]. In effect, if individual variability in obesity treatment remains as high as it is presently, identifying variables that moderate outcomes (i.e., that explain for whom treatment works and under what conditions) will justifiably continue to deserve attention from researchers [2,3]. To date, however, evidence shows that individual weight change cannot be accurately predicted, with only a few variables showing positive results [4,5]. Nevertheless, advances in theoretical formulations regarding the process of weight control [6], improved research methodologies [7], and an increasing number of variables tested as potential predictors [8] suggest further progress is possible.

Among the most valuable applications of valid weight loss prediction models is the early identification of individuals with the least estimated probability of success in a given treatment, who could (and perhaps should) be directed to alternative therapies. Research specifically aimed at studying these overweight/obese persons, who are more resistant to current forms of treatment, would be particularly relevant. Equally important are improvements in the matching between treatments and participants, which are dependent on the measurement of relevant pretreatment variables (i.e., that are found to predict success). More individualized programs have the potential for higher cost-effectiveness and improved overall success rates, by targeting specific areas of concern in selected participants or homogeneous groups [9]. Finally, the development of a valid and comprehensive weight loss readiness questionnaire and its use as a screening tool in obesity treatment are additional foreseeable outcomes of this research [10].

We have previously tested a large number of psychosocial and behavioral variables as predictors of short-term weight outcomes [8]. A number of significant pretreatment correlates of 4-month weight loss were identified, including previous dieting and recent weight changes, self-motivation, weight outcome evaluations, body size dissatisfaction, weight-related quality of life, self-esteem, and exercise self-efficacy and perceived barriers. Because this earlier study was primarily hypothesis-generating, confirmatory results are needed. The goal of the present study was to re-evaluate the predictive value of several of these variables in a different sample of women who underwent a comparable weight reduction program. While our previous work has studied women in the United States (US), the present analysis reports on a group of similarly-overweight/obese Portuguese females. Cross-cultural differences in social norms regarding ideal weights, in the role of physical activity, and in eating habits and relationship with food (e.g. [11]) could have an impact on how individuals respond to obesity therapies and also inform researchers about the role of pretreatment variables (moderators) in treatment success. It should be noted that this study was not designed to evaluate the overall effectiveness of the weight loss program but to analyze predictors of short-term results among participants who displayed highly variable levels of success.

## Methods

### Subjects

Subjects were recruited from the community for a 2-year weight management program through newspaper ads, a website, email messages on listservs, and announcement flyers. Subjects were required to be older than 24 years, be premenopausal and not currently pregnant, have a BMI higher than 24.9 kg/m^2^, and be free from major disease to be eligible for the study. After several orientation sessions, 152 women signed up for the program. During the run-in phase, four women decided not to participate (reporting new time and scheduling conflicts), four did not comply with testing requirements and were excluded, three women found out they were pregnant or decided to attempt pregnancy and were also excluded, and one subject was found ineligible due to medical reasons (untreated hyperthyroidism), leaving a total of 140 women who started the intervention. An initial visit with the study physician ensured that subjects met all medical inclusion criteria. All participants agreed to refrain from participating in any other weight loss program and gave written informed consent prior to participation in the study. The Faculty of Human Movement's Human Subjects Institutional Review Board approved the study.

### Assessments

Weight was measured twice, to the nearest 0.1 kg (average was used) using an electronic scale (SECA model 770, Hamburg, Germany) and height was also measured twice, to the nearest 0.1 cm (average was used). Body mass index (BMI) in kilograms per squared meter was calculated from weight (kg) and height (m). In addition to weight and other morphological and physiological variables assessed, subjects filled out a large psychosocial questionnaire battery prior to the first weekly treatment session. This was conducted in standardized conditions of comfort and silence, with a study technician attending every assessment period. To ensure optimal levels of concentration and avoid overburden caused by long periods of psychometric testing, subjects were required to attend three sessions, each lasting approximately 45 minutes.

Portuguese versions of the Impact of Weight on Quality of Life – Lite (IWQOL-Lite, [12]), Self-Motivation Inventory (SMI, [13]), Rosenberg's Self-esteem/Self-concept (RSE, [14]), Exercise Perceived Barriers (EPB, [15]), and Exercise Self-efficacy (ESE, [16]) questionnaires were used. Details of the original English versions of these instruments are described elsewhere [8]. In brief, the IWQOL-Lite measures weight-specific perceived quality of life on five dimensions of daily life (physical functioning, self-esteem, sexual life, public distress, and work) and it also provides a summary score, which was used in this study. The SMI evaluates a general (i.e., context-unspecific) tendency to persevere, finish tasks initiated, maintain self-discipline, and motivate oneself. The RSE measures a person's self-respect and positive self-opinion. The EPB assesses the extent to which the elements of time, effort, and other obstacles are perceived barriers to habitual physical activity. The ESE measures an individual's belief or conviction that she can "stick with" an exercise program for at least 6 months under varying circumstances, in the dimensions of making time for exercise and resisting relapse. Summary scores for both the EPB and ESE were calculated and used in this study. For all instruments, higher scores indicate higher values for the constructs being measured. Forward and backward translations between English and Portuguese were performed for all questionnaires cited above. Two bilingual Portuguese researchers subsequently reviewed the translated Portuguese versions and minor adjustments were made to improve grammar and readability. In this study, Cronbach's alpha estimates were as follows, for the IWQOL-Lite (0.95, 31 items), SMI (0.88, 40 items), RSE (0.81, 10 items), EPB (0.71, 11 items), and ESE (0.77, 10 items), ensuring acceptable to high internal consistency.

Number of previous diets and weight history variables were taken from a diet/weight history questionnaire developed specifically for this study. Weight outcome evaluations were assessed by 4 questions derived from the Goals and Relative Weights Questionnaire (GRWQ, [17]). Subjects were asked to indicate their "dream" weight, and also what would be their "happy", "acceptable", and "disappointing" weights by the end of the 4-month intervention. Each outcome evaluation was computed as the percentage of pretreatment measured weight. Body size dissatisfaction was assessed by the difference between self and ideal body figures selected from a list of 9 female silhouettes of increasing size [18]. High scores (i.e., larger disparity between self and ideal figure) indicate greater body size dissatisfaction. For multiple-item questionnaires, if a subject failed to correctly fill out at least 75% of all items in a summary/global scale or at least 50% of items in a subscale, the corresponding score was not calculated. However, this did not automatically eliminate a subject from analyses, if other (valid) scores could be used for the same participant.

### Intervention

Subjects attended 15 treatment sessions in groups of 32 to 35 women, for approximately 4 months. Average attendance to the treatment sessions was 83%. Sessions lasted 120 minutes and included educational content and practical application classroom exercises in the areas of physical activity and exercise, diet and eating behavior, and behavior modification [19]. Physical activity topics included learning the energy cost associated with typical activities, increasing daily walking and lifestyle physical activity, planning and implementing a structured exercise plan, setting appropriate goals, using logs and the pedometer for self-monitoring, and choosing the right type of exercise, among many others. Examples of covered nutrition topics are the caloric, fat, and fiber content, and the energy density of common foods, the role of breakfast and meal frequency for weight control, reducing portion size, strategies to reduce the diet's fat content, preventing binge and emotional eating, planning for special occasions, and reducing hunger by increasing meal satiety (e.g., increasing fiber content). Cognitive and behavior skills like self-monitoring, self-efficacy enhancement, dealing with lapses and relapses, enhancing body image, using contingency management strategies, and eliciting social support were also part of the curriculum. The intervention team included two Ph.D.- and six M.S.-level exercise physiologists and dietitians, and one behavioral psychologist. Subjects were instructed and motivated to make small but enduring reductions in caloric intake and to increase energy expenditure to induce a daily energy deficit of approximately 300 kcal. Although weight was monitored weekly, subjects were advised that long-term (i.e., after 1–2 years), not necessarily rapid weight reduction was the primary target. In the first session, participants were informed that reaching a minimum of 5% weight loss at 6 months was an appropriate goal in this program and were subsequently instructed to individually calculate the number of kg that corresponded to.

### Statistical Analysis

Measures of central tendency, distribution, and normality were examined for all psychosocial variables at baseline and for weight at baseline and 4 months. Following intention-to-treat principles and to include psychosocial data from all starting subjects in statistical analysis, the Last Observation Carried Forward (LOCF) method was used for 5 subjects who dropped from the program and could not be reached for testing at 4 months (the five subjects dropped after sessions number 10, 11, 12 [two subjects], and 14); in these cases, the last measured weight, which was assessed weekly for each woman with the same scale as used in laboratory testing, was entered as their final weight. The limitations of this method notwithstanding [20], variations of the LOCF are commonly used in obesity longitudinal trials (e.g., [21]). The very small number of subjects for whom 4-month weight data were imputed, all of which were derived from weights measured late in the program, should result in relatively unbiased results [22]. Furthermore, since a trend toward weight regain is common upon subjects leaving treatment, assuming no further weight change after dropping out works against the study's primary hypotheses, providing additional protection from type I error. One subject was removed from analyses that included weight outcome evaluation variables since her values were markedly lower than values from the rest of the group (i.e., it was considered a data outlier).

Rank-order correlation (Spearman's ρ) was used to estimate the linear relationship between predictors and weight change. All but one among independent variables assessed at baseline displayed a non-normal distribution, warranting the use of this non-parametric technique. The dependent measure was expressed as the difference between baseline and 4-month weight. An alternative way to express weight results is to calculate the "residualized" value for 4-month weight, after the effect of baseline weight is removed (i.e., regressed out in linear regression). This method protects against overcorrection of the post by the pre score when using a subtraction score, and also effectively and completely adjusts this new "change" score for the pretreatment weight value [23]. This variable was also used as a dependent variable in analyses.

Quadratic terms were produced for the two weight outcome evaluation variables, to assess the curvilinear relationship between these measures and actual weight results. Multiple regression analysis was performed to assess the multivariate relationships between the independent variables and weight change. In this regression model, the selected predictors (variables which were significant or approached significance in the bivariate analysis) were forced into the model and the squared semi-partial correlation coefficient was calculated to quantify the unique contribution of each predictor to the variance in the dependent measure [23]. Considering the relatively small subject-parameter ratio (24:1) and in the absence of strong theoretical support for a hierarchical entering of predictors into the model, this a priori (forced) model is preferable to a stepwise model as it minimizes instability in the selection of variables into the model (and in parameter estimation) caused by potential sampling biases [24]. A distribution-based criterion was employed to form three equally numbered groups, split by the two tertiles of weight change. Means of independent variables for the three subgroups were compared by analysis of variance (ANOVA), followed by post-hoc comparisons (Tukey's Honestly Significant Difference test). Type I error was set at 0.05 for all tests. Statistical analyses were completed using the Statistical Package for the Social Sciences (SPSS), version 12.0.

## Results

**Figure 1:**
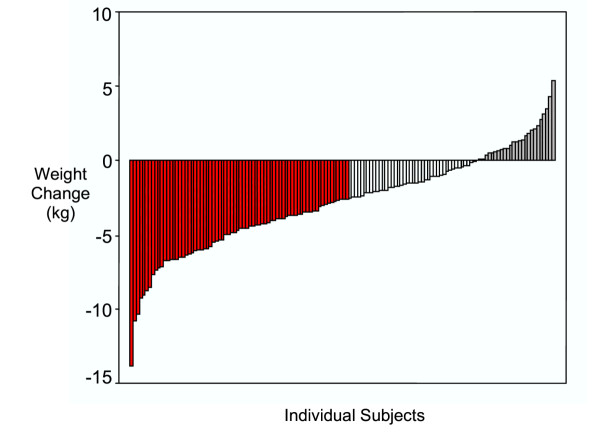
**Individual Weight Change After 4 Months. **Red bars indicate subjects who lost more than 3.3% of their initial weight; grey bars indicate subjects who did not lose weight or who gained weight.

Weight loss data reported in the present study refer to the initial 4 months of a longer trial. After the 4-month phase, subjects were randomly assigned to three distinct long-term interventions. Figure 1 shows individual weight changes for all 140 participants who started the program. Attrition was very low (3.5%) and average weight change was -2.9 ± 3.2 kg (-3.0 ± 3.2 kg, if only the 135 completers are considered). The range for weight change was about 19 kg, a (large) level of individual variability providing an optimal setting to study correlates of weight loss. About 53% of participants lost more than 3.3% of their initial weight (roughly the equivalent of a 5% weight loss after 6 months, in red in Figure 1), thus generally meeting or surpassing the recommended weight loss goals. Eighteen percent of all women (in grey in Figure 1) did not lose, or gained weight after 4 months.

Table 1 shows descriptive statistics for the independent variables and their association with weight change. Fewer previous diets, weight outcome evaluations, and to a lesser degree self-motivation and body image were positively associated with weight loss. When the significance level was adjusted for the number of variables being tested (Bonferroni adjustment, new significance set at 0.005), the number of previous diets and weight outcome evaluations remained significantly correlated with weight results. An additional weight history question, asking whether subjects had lost at least 5 kg in the previous 2 years, was not associated with weight loss at 4 months (t = 0.71, *p *= 0.480, comparing subjects responding "yes" and "no"). Two additional variables from the GRWQ were also analyzed. "Dream" weight (mean ± SD, 98.1 ± 3.9%) was unrelated to baseline-adjusted weight loss (ρ = 0.001, *p *= 0.98) while "disappointing" weight outcome (77.4 ± 7.8% of initial weight) was associated with baseline-adjusted weight loss (ρ = 0.27, *p *= 0.002). Time at current weight, obesity-specific quality of life, self-esteem, and exercise variables were not associated with weight results, before or after adjusting for baseline weight. Significant predictors in the bivariate analysis (Table 1) were entered into a multivariate regression model to predict weight change. Since "happy" and "acceptable" outcome evaluations were highly intercorrelated and represent similar constructs, they were averaged into a single variable for this analysis. All variables entered in the model explained independent shares of the variance in weight loss, before (not shown) and after the inclusion of baseline weight (Table 2). Each predictor caused a significant increase in the model's R^2 ^with weight outcome evaluations explaining the single largest share of the dependent variable. The model accounted for about 24% of the variance in 4-month weight change.

**Table 1:** Correlation Between Pretreatment Variables and Weight Change at 4 Months

	Weight Change			Weight Change^1^					
	n	ρ	*p*	ρ	*p*	Mean	SD	Min	Max
Number of diets in past year	130	0.26	0.002	0.26	0.003	1.2	1.7	0	8
Months at current weight	127	-0.13	0.139	-0.13	0.157	24.1	24.1	0	120
"Acceptable" weight loss (% initial)	134	0.33	<0.001	0.26	0.002	92.7	4.0	77.1	100.6
"Happy" weight loss (% initial)	135	0.27	0.001	0.21	0.015	89.0	4.9	74.9	99.1
Impact of weight on quality of life	138	0.02	0.837	-0.05	0.594	79.5	14.1	37.9	100.0
Self-motivation	135	-0.19	0.030	-0.18	0.036	141.3	17.9	100.0	183.0
Body size dissatisfaction	134	0.09	0.280	0.18	0.038	2.29	0.88	0	5
Self-esteem	131	0.00	0.970	-0.01	0.930	32.4	3.77	24	40
Exercise perceived barriers	138	0.08	0.364	0.08	0.359	29.8	6.29	12	43
Exercise self-efficacy	138	-0.03	0.721	-0.03	0.750	38.3	4.78	25	49

Higher scores indicate higher value for characteristic tested (e.g. higher quality of life, higher self-motivation, higher body size dissatisfaction, more perceived barriers, etc.); Since weight change was coded as baseline weight subtracted to 4-month weight, weight loss is represented by a negative value (thus, a negative correlation coefficient indicates a positive correlation with weight loss). ^1^Four-month weight adjusted for baseline weight.

**Table 2:** Multiple Regression Analysis for 4-month Changes in Weight

	B	t	*p*	Squared semi-partial correlation (%)
Baseline weight	-.069	-2.481	0.015	4.0
Number of diets in past year	.372	2.439	0.016	3.8
Weight outcome evaluations^1^	.235	3.673	<0.001	8.7
Self-motivation	-.040	-2.714	0.008	4.7
Body size dissatisfaction	.755	2.389	0.018	3.7

R^2^_(×100) _= 24.0 (adjusted R^2^_(×100) _= 20.5), SEE = 2.80 kg, F(df,123) = 7.84 (*p *< 0.001); ^1^Average of "happy" and "acceptable" weight outcome evaluations.

**Figure 2:**
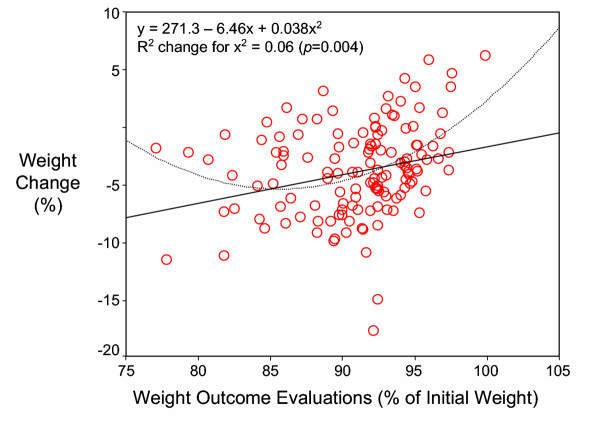
**Relationship Between Weight Outcome Evaluations and Weight Loss. **Dashed line shows curvilinear (quadratic term) and solid line shows linear relationship between weight outcomes evaluations (average of "happy" and "acceptable" values) and weight loss (% of initial). Regression equation includes both linear and quadratic terms and *R*^2 ^*change *refers to the addition of the quadratic term into the model, after the linear term was already in the model.

Weight outcome evaluations were computed as a percentage of participants' initial weight. Thus, the lower this percentage, the more stringent (i.e., more demanding) was a subject's evaluation of her results, and vice-versa. We found significant and positive linear relationships between outcome evaluations and weight loss (Tables 1 and 2), indicating that the more demanding the evaluations of outcomes were at baseline (i.e., the lower the percentage of initial weight), the more weight was later lost (and vice-versa, i.e., the more accepting the evaluation of future weight loss, the less weight subjects lost). However, a visual inspection of these associations suggested that participants on the lower end of the outcome evaluation distribution might not be following the overall group trend. In fact, an additional analysis revealed that, for the whole group, a curvilinear pattern of association described the relationship slightly better than a linear pattern, for both "happy" and "acceptable" outcome evaluations and for the average of the two variables (Figure 2). Quadratic (squared) terms were tested in regression models, following procedures described by Cohen and Cohen [23], and were shown to produce small but significant increases in R^2^, in addition to the non-transformed, linear variables alone. Both linear and curvilinear relationships are depicted in Figure 2. To account for skewness in the weight outcomes data, regression analyses were repeated with the top and bottom 5% of observed values removed from analysis, yielding very similar results (y = 503.9 - 11.5x + 0.065 x^2^; R^2 ^change for x^2 ^= 0.05, *p *= 0.010).

**Figure 3:**
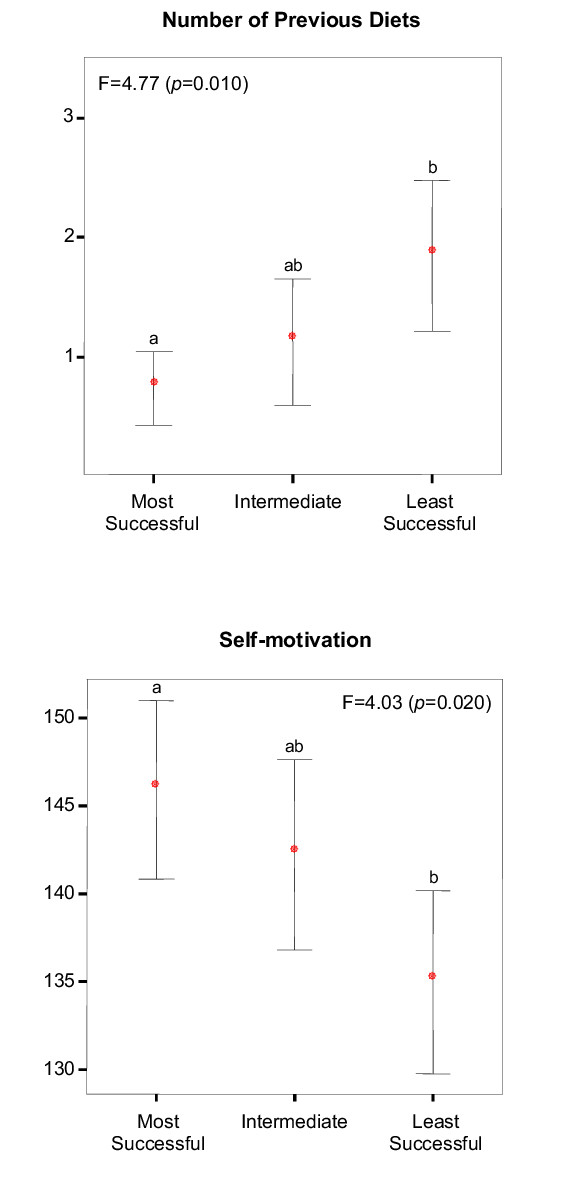
**Comparison of Success Groups for Previous Dieting and Pretreatment Self-motivation. **Groups based on tertiles for 4-month weight loss. F for ANOVA. Error bars show 95^th ^confidence interval. Different letters indicate significant group differences in post-hoc analysis (*p *< 0.05).

To further explore the association of the selected predictors with weight outcomes, subjects were divided into three groups based on tertiles of weight reduction adjusted for initial weight, and baseline psychosocial measures were compared among groups. Significant overall (ANOVA) differences emerged for the number of previous diets and self-motivation, with post-hoc comparisons showing significant mean differences only between the most and least successful groups (Figure 3). Considering the slightly curvilinear relationships observed for the GRWQ variables, it was not surprising that significant differences were not detected between success groups for "happy" (*p *= 0.284) and "acceptable" (*p *= 0.145) weight loss evaluations. Body size dissatisfaction scores were also not different among the three groups (*p *= 0.432). Table 3 shows the frequency of previous diets reported by each success group in more detail. Of all subjects reporting no diets initiated in the previous year, only 17% finished in the least successful groups. Conversely, of the 20 subjects reporting 3 or more recent diets, only 3 (15%) finished within the most successful group. Ten women reported having initiated 4 to 8 diets in the previous year, none of whom finished the 4-month program in the group of women losing the most weight.

**Table 3:** Frequency of Diets Initiated in the Previous Year, by Weight Loss Success Group^1^

	*Most Successful*			*Intermediate*			*Least Successful*		
Number of diets	Freq.	%	Cum.%	Freq.	%	Cum.%	Freq.	%	Cum.%
0	25	58	58	24	52	52	10	25	25
1	7	16	74	10	22	74	14	34	59
2	8	19	93	6	13	87	6	15	73
3+	3	7	100	6	13	100	11	27	100
Weight loss (kg)									
Mean		-6.3			-2.7			0.3	
SD		2.1			1.0			1.7	

^1^Groups defined based on tertiles of 4-month weight loss adjusted for baseline weight.

## Discussion

This study aimed at reexamining the association between several pretreatment individual characteristics and success in short-term behavioral weight reduction, in overweight and moderately obese women. Ten variables which had previously been shown to predict weight change [8] were analyzed in a separate sample, using a comparable research methodology. Previous dieting, self-motivation, and body image showed significant effects as predictors and in the expected direction of relationship. Participants' evaluations about possible weight outcomes were also significantly associated with weight loss in the present study, although in a direction opposite than what was hypothesized; more stringent evaluations of outcomes had predicted worse outcomes in US women [8] while the reverse was observed in Portuguese women for whom more accepting attitudes towards weight loss were associated with smaller weight changes. Earlier results for exercise, quality of life, self-esteem, and also for some variables related to weight history (time at current weight and large recent weight losses), were not confirmed in the present study.

To date, the majority of research on the treatment of overweight and obesity has focused on assessing overall treatment efficacy (expressed as mean group weight change, number of individuals reaching some marker of success, etc.) and analyzing which programs work best, typically using randomly-assigned experimental treatment groups [25-27]. By contrast, much less research has been undertaken to investigate the *mechanisms *(mediating variables) by which treatments affect subjects, and *for whom *treatments are most effective (i.e., individual moderators). The potential benefits of studying moderators and mediators of outcomes within the behavioral and social sciences, including for physical activity, diet, and weight control are well described in the literature [28-30]. The identification of such variables open the way to a new generation of interventions, characterized by a higher level of individualization and overall efficacy, both by targeting those individuals more likely to succeed and through an increased focus on those mediators (treatment-related, environmental, and individual factors, and critical interactions among them) more clearly associated with outcomes [7]. Nevertheless, empirically-derived hypotheses for the role of moderators and mediators in the treatment of obesity remain scant, particularly for psychosocial variables. As a contrasting example, sufficient evidence was already available in the alcohol prevention field in the early 1990's for a large multi-center trial to be funded and carried out, aimed at testing the interaction between treatment modality and a considerable number of individual predictors/moderators such as cognitive impairment, conceptual level, motivation, social support, and patient typology [31].

In the present study and in other trials [32-36], previous dieting and weight loss attempts have emerged as reliable negative predictors of weight loss. One explanation is that the subset of women reporting more frequent dieting contains a disproportionally high number of individuals who are, for some reason, more resistant to weight control. Despite evidence showing that many individuals are successful even after many previous failed attempts [37,38], it is possible that some subjects in research-based obesity treatment programs see those programs as just one more among many solutions they have tried and failed at before, and thus are more prone to low self-confidence and impaired motivation. Frequent restriction of eating, implied in the question "how many *diets *have you started...?", could also be a marker for more extreme dieting behaviors that may not be sustainable after the initial boost of motivation [39]. This could also increase the probability for weight rebound. More studies are needed to investigate the mechanisms through which previous dieting affects weight control, a consisting finding in the literature. The present report also provides indication that a threshold may exist (3–4 number of diets in the previous year) which is associated with a marked reduction in the likelihood of success.

Four earlier reports have examined the role of self-motivation as a predictor of weight loss [8,36,40,41] while one additional study used a general self-efficacy questionnaire worded similarly to the SMI [42]. The related construct of autonomy-oriented motivation (defined as a motivation style more related to a persons' own interests and values and less controlled by external events) has also been evaluated as a predictor [43]. With one exception [41], evidence has supported the notion that high pretreatment levels of self-motivation and an autonomy-oriented motivation are beneficial traits for subsequent weight loss. The SMI has also been shown to correlate with eating variables during weight loss [44] and to predict exercise behavior [13]. Contrary to earlier observations in US women [8,36], exercise-related variables did not predict weight loss in the present analysis. That is, while the more general personality attributes related to motivation and efficacy were stable predictors of outcomes in weight loss across studies, the moderating role of exercise self-efficacy and exercise perceived barriers (time, effort, etc.) did not translate well from the US to the Portuguese data set. Cross-national differences such as distinct levels of social awareness for exercise or differences in level of knowledge, past adoption levels, and/or perceived competence regarding exercise and physical activities, all of which may have influenced answers to the exercise questionnaires, are possible explanations for these differences.

This study is among only a few that have analyzed associations between the Goals and Relative Weights Questionnaire and subsequent weight loss. Interestingly, marked differences emerged between the present and two previous analyses [8,36]. Portuguese women with more modest weight outcome evaluations were less likely to lose weight, while in US women the opposite was observed, that is, more stringent (demanding) evaluations of possible results were predictive of poorer results. Evidence for a significant effect of outcome expectancies on weight control is extremely relevant in the context of realistic versus unrealistic expectations for weight loss [45-47]. Excessively optimistic expectations are common in US treatment-seeking obese samples [17], for whom a great value is typically placed on reaching desired weights [48]. By contrast, Portuguese women, perhaps because their are comparatively less exposed to external pressures to be thin and/or because they belong to a culture where optimism is less valued than in the US, were less likely to produce very demanding weight-related evaluations. Accordingly, we have recently reported that Portuguese women do, on average, state overall less stringent evaluations of weight loss outcomes at baseline than their American counterparts [49]. This being the case, one hypothesis for the divergent associations for US and Portuguese samples is that, when a broad population is considered, the expectations-outcomes relationship is indeed curvilinear (with an yet-undetermined nadir or interval representing the more favorable goals/expectations) and that Portuguese women predominantly fall on the right (more conservative) side of the distribution while US subjects better represent the left side (more stringent).

In the present study, it appeared that the weights participants would find acceptable/happy were associated with weight loss (i.e., more "optimistic" outcome evaluations, more weight loss) until a certain threshold was reached, somewhere around 85 to 90% of initial weight (10–15% weight loss); for women reporting outcome evaluations below that level no further benefit was apparent. One previous study has shown that women with more modest absolute weight loss goals were more likely to achieve their goals, and that those who achieved their weight goals had better weight maintenance after 2.5 years; however, desired weight loss did not directly predict actual weight loss [50]. Positive expectations expressed as a higher reported likelihood of reaching goal weight predicted larger short-term weight loss in subjects who showed lower level of fantasizing and daydreaming about beneficial consequences of large weight loss [51]. Other studies have shown larger weight loss goals to positively predict weight loss [41,52] and in one other case goals had small predictive value [53]. Collectively, previous results and those we now report suggest that positive *and *moderate expectations/outcome evaluations foretell the best overall results, particularly if accompanied by a high sense of self-assurance [52].

It should be noted that variables originating from the GRWQ are closely related but are not equivalent to the construct of outcome expectancies (the belief that certain actions will lead to the projected results [54]) or to weight loss goals. The GRWQ seems to partially measure an actual prediction of outcomes by the participant, similar to a general self-efficacy expectation (e.g., *how much weight do you think you will lose by the end of this program?*), while simultaneously tapping into a more attitudinal facet towards a person's weight and weight loss (*how happy/accepting/disappointed would you feel at certain levels of weight loss?*). To some extent, the latter could measure idealization of body weight and perceived importance of body weight and shape for self-esteem and well-being. Therefore, it is possible that moderate or "realistic" weight outcome evaluations (i.e. not too accepting but also not excessively stringent) are the most beneficial and indeed reflect a good balance between a sufficient and necessary sense of self-efficacy *and *low to moderate levels of thin-ideal internalization, a variable which has been shown to be a positive risk factor for body dissatisfaction, negative affect, and eating disorders [55,56].

Women reporting a larger discrepancy between self and ideal body figures, which indicates a higher body size dissatisfaction [18], were less likely to lose weight. In a previous report, the same self-ideal measure correlated similarly with short-term results, while two other measures of body image showed comparable, albeit non-significant trends [8]. Pretreatment scores in the body dissatisfaction scale of the Eating Disorders Inventory, a measure of psychological concern and dislike about one's body shape and size [57], has also been negatively associated with weight loss in two other behavioral weight loss programs [58,59]. These relationships may be explained by the negative association of body image with mood and psychological impairment [60], and also by the disappointment and lack of self-worth and self-confidence following previous failed attempts to change weight and body shape [6]. Although self-esteem did not predict outcomes, we observed significant correlations between body size dissatisfaction and self-esteem (ρ = -0.18, *p *= 0.042), the number of previous diets (ρ = 0.22, *p *= 0.013), and weight-related quality of life (ρ = -0.37, *p *< 0.001). Rapid and concurrent improvements in body image and eating behavior (e.g., reduction in binge episodes) have been observed after surgery-induced thinning [61], clearly suggesting a close link between attitudes towards one's body and weight control behaviors. Body image therapy has also been shown to reduce concern with food, in the context of a behavioral weight control trial [62]. Despite the sound theoretical rationale and supportive body of evidence, a note of caution must be made regarding the multidimensionality of the body image construct [63] and the proliferation of assessment instruments for body image. Although they are typically intercorrelated [60], different body image scales should be interpreted separately as they may result in different patterns of association with weight loss [8,58].

Strengths of this study are the a priori selection of variables to be analyzed as predictors, a unique population (Portuguese women), and the very low dropout rate. Limitations include a moderately-sized sample considering the known measurement error associated with questionnaire psychological assessments, the fact that some of the scales used still lack well-established validity, and the absence of a control or comparison group.

## Conclusions

Several pretreatment variables were re-evaluated as predictors of short-term weight loss in women. Previous dieting, low self-motivation, and body size dissatisfaction were confirmed as negative predictors of weight outcomes, while the relationship of outcome evaluations with weight reduction suggested a negative and curvilinear pattern, with positive but not excessively demanding evaluations presaging the best results. These data regarding people's outcome evaluations prior to weight loss may have important clinical implications [64] and are the first evidence for such a pattern of association; thus, they await replication in other samples. Additionally, treatment decisions based on level of previous dieting (alone or included in comprehensive prediction models) may be possible in the near future, at least for overweight and moderately obese women. The more consistent predictors from this and previous studies (e.g., [8,42,59]) can and should be used in future hypothesis-testing studies of moderators of weight loss. Finally, this study highlights the fact that behavioral and psychological prediction models may, to some extent, be specific to a particular culture [65]. Hence, it is likely that some variables will emerge as moderators (and mediators) of obesity treatment in some, but not all cultures, while others will be proven as more universal correlates of success.

## Competing Interests

None declared.

## Authors' Contributions

PJT conceived the study, led the implementation team, performed most statistical analysis, and drafted the manuscript. ALP participated in the study's implementation, in statistical analysis, and was responsible for all psychometric assessments. TLB, SSM, CSM, and JTB were actively involved in the study's implementation and in data collection. AMS participated in the study's implementation and collected all body habitus data. LBS is the principal investigator in the research trial and contributed to the final version of the manuscript. All authors read and approved the final manuscript.
